# Fluorescent sensing platform for low-cost detection of Cu2+ by coumarin derivative: DFT calculation and practical application in herbal and black tea samples

**DOI:** 10.3906/kim-2004-63

**Published:** 2020-08-18

**Authors:** Tahir SAVRAN, Abdurrahman KARAGÖZ, Şükriye Nihan KARUK ELMAS, Duygu AYDIN, Furkan ÖZEN, Kenan KORAN, Fatma Nur ARSLAN, Ahmet Orhan GÖRGÜLÜ, İbrahim YILMAZ

**Affiliations:** 1 Department of Chemistry, Kamil Özdağ Science Faculty, Karamanoğlu Mehmetbey University, Karaman Turkey; 2 Department of Mathematics and Science, Faculty of Education, Akdeniz University, Antalya Turkey; 3 Department of Chemistry, Faculty of Science, Fırat University, Elazığ Turkey

**Keywords:** Coumarin, fluorescent sensor, copper, DFT, tea samples

## Abstract

A fluorogenic probe based on a coumarin-derivative for Cu^2+^ sensing in CH_3_CN/H_2_O media (v/v, 95/5, 5.0 μM) was developed and applied in real samples. 3-(4-chlorophenyl)-6,7-dihydroxy-coumarin (MCPC) probe was obtained by synthetic methodologies and identified by spectral techniques. The probe MCPC showed remarkable changes with a “turn-off” fluorogenic sensing approach for the monitoring of Cu^2+^ at 456 nm under an excitation wavelength of 366 nm. The response time of the probe MCPC was founded as only 1 min. The detection limit of the probe MCPC was recorded to be 1.47 nM. The binding constant and possible stoichiometric ratio (1:1) values were determined by Benesi-Hildebrand and Job’s plot systems, respectively. The mechanism of the probe MCPC with Cu^2+^ was further confirmed by ESI-MS and FT-IR analyses, as well as supported by theoretical calculations. Furthermore, the probe MCPC was successfully employed for the practical applications to sense Cu^2+^ in different herbal and black tea samples. The proposed sensing method was also verified by ICP-OES method.

## 1. Introduction

The rapid industrialization has caused heavy metal pollution which is gradually becoming a critical issue over the past three decades. This pollution is considered as a major threat, because heavy metal ions lead to serious problems for the ecological environment and human health [1]. Among the commonly encountered heavy metals of concern, the Cu^2+^ is the third most plentiful trace cation in the clay as well as in numerous livings, and it acts an essential role in numerous biological reactions. Cu^2+^ acts a vital role in the connective tissue growth, bone and blood formation as one of the physiological processes of organisms [2], it is also placed in the cornea and mostly in the brain [3]. However, it has been reported that excess intake of Cu^2+^ causes adverse health effects in the body such as gastrointestinal diseases and it damages the kidneys and liver [1,4]. Also, Cu^2+^ catalyzes the creation of reactive oxygen species which can harm basic biological molecules. According to various literatures, the toxicity of Cu^2+^ has linked to severe neurological diseases [5], Indian childhood cirrhosis (ICC) and prion diseases [2,4,6,7]. World Health Organization (WHO) has reported that the optimum intake of Cu^2+^ for adults should not go above the level of 10–12 mg.day^−1^ [8]. For this reason, the monitoring of Cu^2+^ in a variety of drinking and food samples has been a vital issue for the protection of human health [7]. Thus, the development of reliable analytical procedures for Cu^2+^ sensing is still a critical research topic [9].

The reported methods for monitoring Cu^2+^ include inductively coupled mass-atomic emission spectrometry (ICM-AES), capillary electrophoresis (CE), atomic absorption spectrometry (AAS), voltammetry and inductively coupled plasma-mass spectrometry (ICP-MS) [10]. These techniques involve expensive analysis systems, troublous analysis procedures, and also need excessive use of samples [11]. In contrast, the fluorescence spectroscopy is currently taken into account as a powerful method for the recognition of Cu^2+^ owing to its real time, low cost, rapid and non destructive sensing with high selectivity and sensitivity [12] and easy operation. Recently, many fluorescent chemosensor studies for the recognition of Cu^2+^ have been reported [5,13,14]. To this end, various fluorescent probes based on rhodamine [15], naphthalene [16], BODIPY [17], fluorescein [18], N-aminophthalimide [19], thiophene [20], indole [21], pyrene [22], carbazole [23], cyanine [24] and coumarin [15] derivatives have employed to determine Cu2+ in several samples. Among these derivatives, coumarin-based molecules are powerful and highly adjustable fluorescent platforms for the selective and precise measurements of Cu^2+^ in drinking water, food and beverage samples [12].

Herein, a “turn-off” fluorogenic probe based on a coumarin-derivative, 3-(4-chlorophenyl)-6,7-dihydroxycoumarin, probe MCPC, for Cu^2+^ sensing in CH_3_CN/H_2_O media (v/v, 95/5, 5.0 μM) was prepared and employed to various herbal and black tea samples. Coumarin compounds have been commonly used to be a florescent sensor thanks to its moderately high water solubility, high fluorescence quantum (Φ) yield and chemical stability, and also its large Stoke’s shift. In addition, coumarin derivatives are less toxic to the livings and eco-friendly compounds [9]. The synthesized probe MPCP was successfully identified by spectral techniques such as ^1^H-NMR and 13 C-ATP-NMR, as well as FT-IR. The theoretical computations were also done for the optimized geometric features.

## 2. Materials and methods

### 2.1. Chemicals and instrumentation

All chemicals used herein were of spectroscopic grade and obtained from Merck KGaA (Darmstadt, Germany) and VWR International B.V. (Amsterdam, Netherlands). Ultrapure water was acquired from Milli-Q® 7003/05/10/15 machine (Water purification system, Merck KGaA, Darmstadt, Germany). Solutions of different metal ions (Hg^2+^, K^+^, Mn^2+^, Cu^2+^, Ba^2+^, Cd^2+^, Co^2+^, Ca^2+^, Zn^2+^, Mg^2+^, Na^+^, Fe^2+^, Sr^2+^, Pb^2+^, Fe^3+^ and Al^3+^) (10 mM, perchlorate salts in acetonitrile) were prepared as a stock solution.

A Bruker DPX 400 MHz spectrometer (Bruker Corp., Billerica, MA, USA) was employed to measure ^13^C-APT-NMR and ^1^H-NMR spectra. A Mass spectrometer (Bruker Daltonics-Microflex^TM^, Bruker Corp.) was used to record electro spray ionization-mass spectra (ESI-MS). Infrared spectra were taken on a FT-IR spectrometer (Spectrum 100, Perkin Elmer Inc., Wellesley, MA, USA). A fluorescence spectrophotometer (Cary Eclipse, Agilent Tech., Santa Clara, CA, USA) was employed to record fluorescence spectra at rt. As a reference method, the amount of Cu^2+^ in herbal tea samples was analyzed by an inductively coupled plasma-optical emission spectroscopy (ICP-OES) (720, Agilent Tech.). For the pH measurements and centrifugation procedures, a pH meter (Mettler Toledo, Zaventem, Netherlands) and Universal 320R centrifuge system (Andreas Hettich GmbH & Co. KG, Tuttlingen, Germany) were used, respectively.

### 2.2. Fabrication of the probe MCPC

Probe MCPC, 6,7-dihydroxy-3-(4-chlorophenyl)coumarin, was successfully synthesized under microwave irradiation procedure with solvent free conditions according to the reported method in the literatures [25–27]. The synthesis protocols and characterization data were presented in Supplementary Information file in detail.

### 2.3. Fluorescence spectroscopic studies of the probe MCPC towards Cu^2+^ sensing

Probe MCPC was dissolved in acetonitrile as a stock solution and 10 mM of this was adjusted to 5.0 μM concentration with the mixture of CH_3_CN/H_2_O (v/v, 95/5). The titration experiments were performed to achieve the relationship between emission intensities and Cu^2+^. For the fluorescence titration experiments, the different amounts of the cations (0–3.0 equiv) were added to 3000 μL of the probe solution. Spectra were taken at room temperature and recorded at λ_em_ = 456 nm (λ_ex_ = 366 nm, 5 nm slit width).

The LOD of the probe MCPC was calculated from the equation of 3σ/k (slope value) [28–31]. To analyze the effect of the possible competing cations on the sensing ability of probe MCPC to Cu^2+^, suitable concentration of Cu^2+^ (3.0 equiv) was added into probe MCPC solution to generate MCPC-Cu^2+^ complex, afterwards each of the other cation solutions was added into the MCPC-Cu^2+^, respectively. The emission intensities of the MCPC-Cu^2+^ complex were measured before and after adding other cations. The possible stoichiometric ratio between the probe MCPC and Cu^2+^ in MCPC-Cu^2+^ system was found by using Job’s method calculation [32].

### 2.4. Studies based on DFT

Theoretical calculations were performed for the probe MCPC and MCPC-Cu^2+^ complex by the DFT / B3LYP/6-31 g (d) method with Gaussian 09 software (Gaussian, Inc., Wallingford, CT, UK) and accompanying graphical interface program GaussView 5.0.8 [33–37]. The LanL2DZ basis set for the effective potential set for Cu, and the 6-311G basis set was used for C, H, O atoms. To obtain the HOMO and LUMO energy levels, DFT analyses were performed based on the optimized geometries.

### 2.5. Detecting Cu^2+^ in tea samples by fluorometric sensing and ICP-OES

Eleven kinds of herbal tea leaves (green tea, white tea, sage tea, fennel tea, daisy tea, rose hip tea, ginger tea, mint tea, apple tea, linden tea and green tea mixture with rose) and 5 kinds of black tea leaves (black tea samples without aroma and black tea samples with bergamot aroma) were bought from super markets and herbs shops. 2.0 g of each samples were homogenized, individually placed into the boiled ultrapure water (50.0 mL) and infused/waited approximately for 15 min. Prepared tea solutions were placed in a shaker for 20 min, and then they were centrifuged at 10.000 rpm for 5 min. Final solutions were cleaned with a membrane filter (0.45 μm pore-size) and then the amounts of Cu^2+^ in teas were identified by the probe MCPC and ICP-OES systems. For fluorometric sensing analysis, probe MCPC solution was freshly prepared in CH_3_CN/H_2_O media (v/v, 95/5, 5.0 μM). 3000 μL of probe MCPC solution was put into the quartz-cuvette, and then the spectra were measured at rt (λ_ex_ = 366 nm, λ_em_ = 456 nm, 5 nm slit width). Then, 15 μL of each tea samples individually put into the probe MCPC, and the spectra were recorded by the same procedure. Finally, this solution (tea sample + probe MCPC) was spiked with 15 μL of diverse amounts of Cu^2+^ (0.1 and 0.2 μM) (standard addition method) and spectra were obtained. For ICP-OES analysis, the solution of tea samples were acidified with HNO_3_ (v/v, 1%) in the 10 mL of centrifuge tubes. pH value of these solutions were adjusted to 9 with a buffer solution (NH_3_/NH_4_Cl), and just after 15 min of waiting time, all teas were centrifuged at 10.000 rpm (~5 min). 0.5 mL of d-HNO_3_ solution was added to the bottom layer in the tubes and this layer was diluted to 2500 μL with ultra-pure water. Before ICP-OES analysis, the sample solution was cleaned with a membrane filter (0.45 μm pore-size) to eliminate possible contaminations. The operating conditions of ICP-OES method were presented in Table S1. The analyses were done in triplicate and outcomes were illustrated as mean ±std.

## 3. Results and discussion

### 3.1. Design of the probe MCPC

**Scheme 1:**
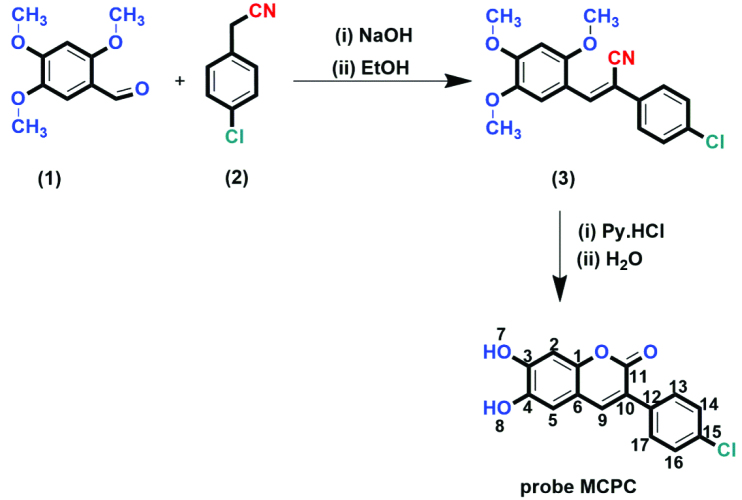
Synthetic scheme of the probe MCPC.

The probe MCPC was prepared under microwave irradiation with solvent free condition by the reaction of compound (3) with pyridinium hydrochloride (85% yield) (Scheme 1), and it was well-verified by ^1^H-NMR, FT-IR and ^13^C-ATP-NMR analyses (Figures S1–S6).

As seen in FT-IR spectrum of the probe MCPC, the peaks appeared at 3197 and 3398 cm^−1^ were revealed the presence of -OH and also, -C = O peak in the coumarin ring was appeared at 1660 cm^−1^ (Figure S4). Moreover, the proton NMR for 2, 5, and 9 protons gives singlet peaks at 6.80, 7.08, and 8.16 ppm, respectively. In addition, protons 13 and 14 give doublet peaks at 7.51 and 7.74 ppm. The -OH proton peaks are observed at 9.56 and 10.34 ppm belong to H^7^ and H^8^. Characteristic functional group conversions show that the formation of probe MCPC was successfully achieved.

### 3.2. Fluorogenic response of the probe MCPC towards Cu^2+^ sensing

**Figure 1:**
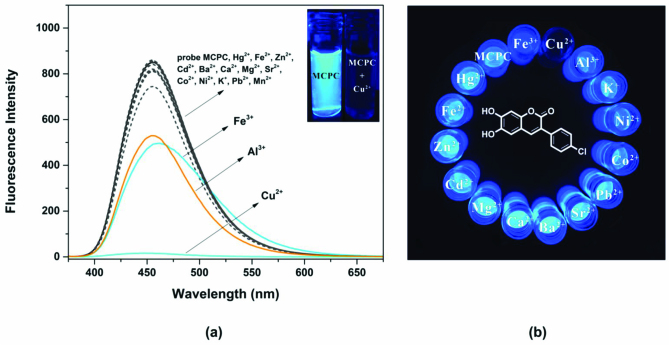
(a) Spectra of probe MCPC in the presence of competing ions (3.0 equiv), and (b) fluorescence colors of MCPC in presence of competing cations in CH_3_CN/H_2_O media (v/v, 95/5) (5.0 μM) (λ_ex_ = 366 nm, λ_em_ = 456 nm).

To study the fluorogenic sensing ability, the response of probe MCPC towards a series of various cations (Hg^2+^, K^+^, Mn^2+^, Cu^2+^, Ba^2+^, Cd^2+^, Co^2+^, Ca^2+^, Zn^2+^, Mg^2+^, Na^+^, Fe^2+^, Sr^2+^, Pb^2+^, Fe^3+^ and Al^3+^) was investigated in CH_3_CN/H_2_O media (v/v, 95/5, 5.0 μM) (Figure 1). The sensing property of probe MCPC was tested in the presence of possible competing ions at a fluorescence emission wavelength of 456 nm. As illustrated in Figure 1, only Cu^2+^ caused a diverse intensity change and emission intensity decreased sharply, whereas the probe MCPC did not give fluorogenic response towards other cations. None of the other cations caused remarkable impact on the intensity of probe MCPC. The probe MCPC exhibited only a small intensity change in the presence of Al^3+^ and Fe^3+^. Thus, we concluded that the probe MCPC has good selectivity toward Cu^2+^ compared to other potential cationic species.

#### 3.2.1. Competitive experiments

**Figure 2:**
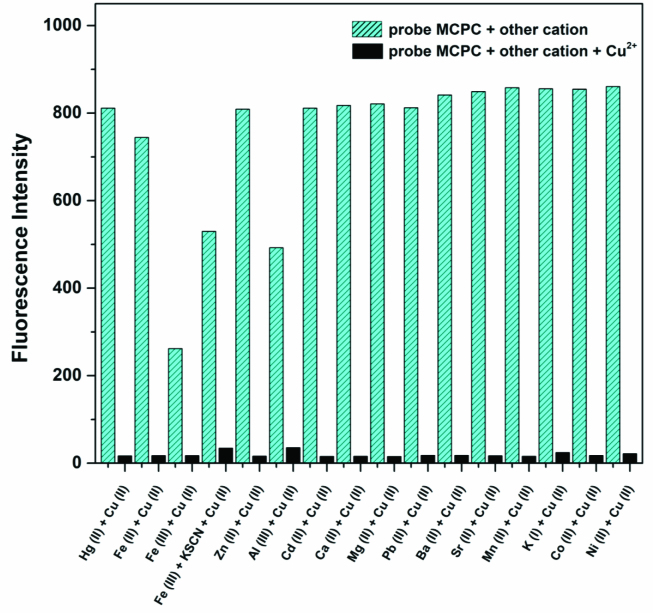
Competitive selectivity of probe MCPC (5.0 μM) toward Cu^2+^ in the presence of competing ions in CH_3_CN/H_2_O media (v/v, 95/5, 5.0 μM) (3.0 equiv, λ_ex_ = 366 nm, λ_em_ = 456 nm).

To determine the practical efficacy of probe MCPC as a fluorogenic chemosensor for Cu^2+^ monitoring, the competitive experiments were performed with various cations (3.0 equiv) (Hg^2+^, K^+^, Mn^2+^, Cu^2+^ , Ba^2+^ , Cd^2+^ , Co^2+^ , Ca^2+^ , Zn^2+^ , Mg^2+^ , Na^+^, Fe^2+^ , Sr^2+^ , Pb^2+^ , Fe^3+^ and Al^3+^) in CH_3_CN/H_2_O media (v/v, 95/5, 5.0 μM) (Figure 2).

The emission intensity of probe MCPC was quenched with Cu2+ in the presence of other ions and there was no interference with other cations except Fe3+ (Figure 2). This cation could be easily masked by potassium thiocyanate (KSCN); hence it has a little impact on the selectivity of probe MCPC toward Cu2+ . That is to say, probe MCPC could be employed as an excellent selective sensor for the monitoring of Cu2+ .

#### 3.2.2. Sensivity experiments

**Figure 3:**
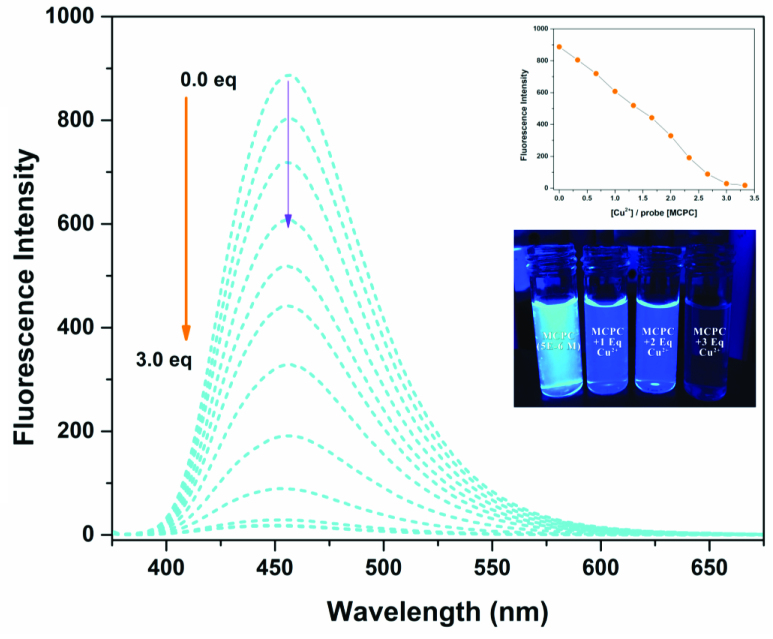
Spectral changes of probe MCPC upon the adding of Cu^2+^ (0 to 3.0 equiv) in CH_3_CN/H_2_O media (v/v, 95/5, 5.0 μM) (λ_ex_ = 366 nm, λ_em_= 456 nm).

Fluorescence titration study was performed to investigate the quantitative interaction features of probe MCPC toward Cu^2+^ (Figure 3). Upon the adding of Cu2+ (0 to 3.0 equiv), probe MCPC showed a gradual emission decreasement up to the constant equiv value, 3.0 equiv. Due to the complexation reaction between the probe MCPC and Cu^2+^, the quenching phenomena could occur via the heavy atom & paramagnetic effect [38] and CHEQ (chelation enhancement quenching effect) [39] mechanisms. Therefore, the probe MCPC could be used as an outstanding “turn-off” fluorogenic sensor for Cu^2+^ monitoring.

#### 3.2.3. Response time experiments

As well, the time dependence of the probe MCPC (5.0 μM) to Cu^2+^ (3.0 equiv) was studied (Figure S7) in CH_3_CN/H_2_O media (v/v, 95/5, 5.0 μM) to decide the reaction time for the MCPC-Cu^2+^. The intensity of probe MCPC reached a maximum/stabile value within approximately 1 min; hence this result showed that probe MCPC could be used as a fluorogenic chemosensor for the rapid sensing of Cu^2+^.

#### 3.2.4. ESI-MS and Job’s plot results

**Figure 4:**
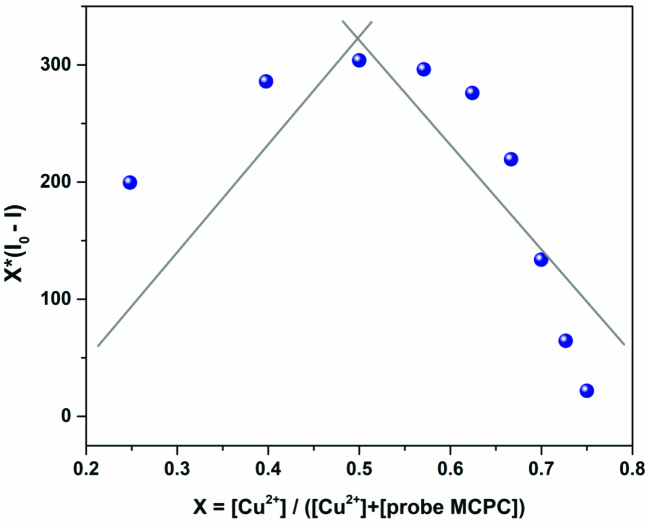
Job’s plots of MCPC-Cu^2+^ complex in CH_3_CN/H_2_O media (v/v, 95/5, 5.0 μM) (λ_ex_ = 366 nm, λ_em_ = 456 nm).

**Figure 5:**
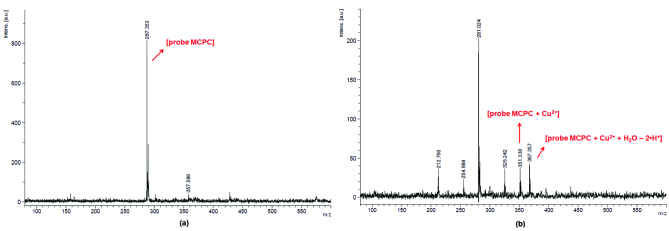
ESI-MS spectra of the probe MCPC and MCPC-Cu^2+^ system.

To consider the binding mode/stoichiometry of probe MCPC toward Cu^2+^, Job’s plot [32] (Figure 4), as well as ESI-MS analysis recorded in different matrixes and modes (Figures 5a and 5b) were carried out. The results of these analyses showed the stoichiometric ratio of MCPC-Cu^2^+ complex as 1:1.

**Scheme 2:**
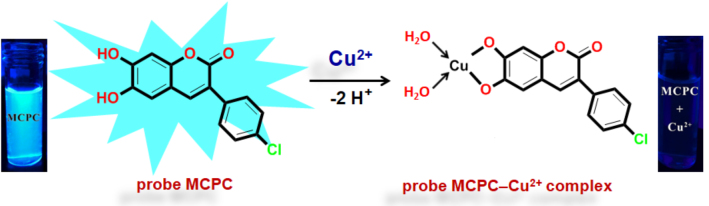
Possible binding mechanism of the probe MCPC upon adding of Cu^2+^.

As illustrated in Figure 5, the peaks at 288.00 [m/z], 351.50 [m/z] and 367.50 [m/z] can be attributed to [probe MCPC], [probe MCPC + Cu^2+^] and [probe MCPC + Cu^2+^+ H_2_O - 2•H^+^], respectively, which demonstrated that probe MCPC complexed with Cu^2+^ in a ratio of 1:1. The content of water in the MCPCCu^2+^ system can be seen from the ESI-MS, as well from FT-IR spectrum (Figure S8). The complexation process was recognized between Cu^2+^ and two protons belong to the hydroxyl groups (Scheme 2).

**Figure 6:**
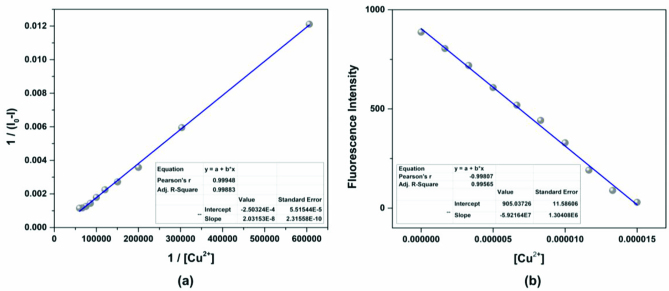
(a) Benesi-Hildebrand curve of 1/[Cu^2+^] vs 1/(I-I_0_), and (b) the calibration curve of the quantitative association between the intensities of probe MCPC and the amount of Cu^2+^ (λ_ex_ = 366 nm, λ_em_ = 456 nm).

On the basis of 1:1 stoichiometry, the binding constant of the MCPC-Cu^2+^ system was computed from the Benesi-Hildebrand equation [40], and it was found to be 1.23 ×10^4^ M^−1^, which inferred that the stability of MCPC-Cu2^+^ complex was very high (Figure 6a). The calibration curve of the quantitative association between the intensity and amount of Cu^2+^ was acquired with an excellent linear range (y = 5.92 ×10^7^ x − 905.04, R^2^ = 0.9957) at nanomolar levels (Figure 6b). The detection limit value of probe MCPC for Cu^2+^ was calculated as 1.47 nM (3σ / slope) [28,29]. The sensing properties of the probe MCPC are comparable to those of other reported literatures which are include cumarin-based fluorescent probes for Cu^2+^ (Table 1). When this study is compared to our previous coumarin based fluorescent sensor studies [9,12], it is obvious that this study is more sensitive than others in terms of the detection limit value as a result of the substituent effect in the structure. Although the flour atom in the previous study is attached to the benzene ring through an aliphatic carbon, the chlorine in this study is directly connected to the benzene and has greater ability to attract the shared pair ofelectrons. Therefore, the acidic behaviors of the hydrogen of hydroxyl group increases, since chlorine in benzene ring demonstrates as well as resonance and inductive effect withdraws electrons while the delocalization of lone pair supplies electron density towards the ring. This system enables us the usability of this sensor for sensing of the toxic Cu^2+^ at lower concentrations. The electron-donating ability of F_3_C^−^, H_3_C^−^, Cl^−^ is examined, it is found that F_3_C^−^ <H_3_C^−^<Cl^−^ and the abilities are directly proportional to the obtained detection limit values (24.5 nM <5.13 nM <1.47 nM). As a consequence, the obtained results showed that the probe MCPC had an excellent sensitivity toward Cu^2+^ sensing, as well as a good linearity for quantitative detections.

**Table 1:** Relative work of the sensing performance of the probe MCPC with some coumarin-based probe studies.

Probe	Solvent system	LOD (M)	Binding constant (Ka, 1/M)	Sample application	Ref.
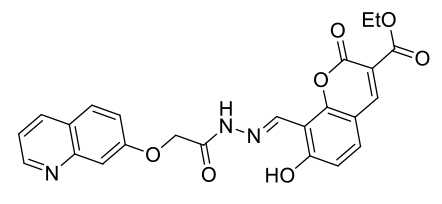	Na_2_HPO_4_-citric acid buffer (10 mmol/L)	256.00 ×10^−9^	1.75 ×10^6^	Yes	[41]
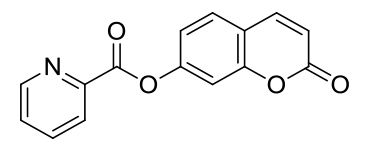	Buffered water solution (10 mM Tris-HCl, containing 1% DMSO)	35.00 ×10^−9^	-	Yes	[42]
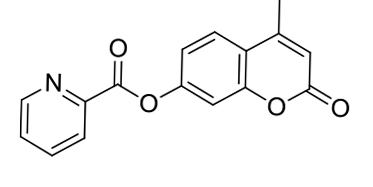	PBS buffer solution /DMSO (v/v, 3/1)	62.00 ×10^−9^	-	Yes	[10]
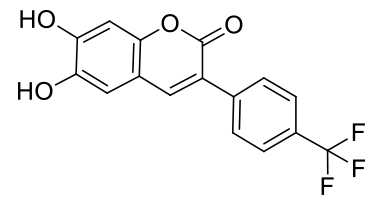	CH_3_CN/H_2_O (v/v, 95/5)	24.50 ×10^−9^	9.00 ×10^4^	Yes	[9]
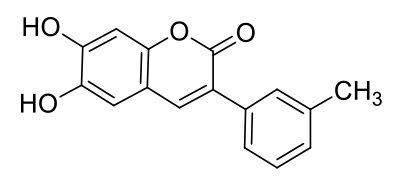	CH_3_CN/HEPES (v/v, 95/5)	5.13 ×10^−9^	8.80 ×10^5^	Yes	[12]
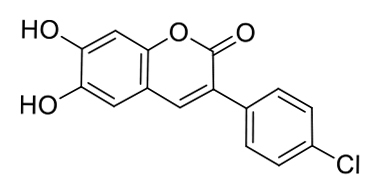	CH_3_CN/H_2_O (v/v, 95/5)	1.47 ×10^−9^	1.23 ×10^4^	Yes	this work

### 3.3. DFT calculations of the probe MCPC and MCPC-Cu^2+^complex

**Figure 7:**
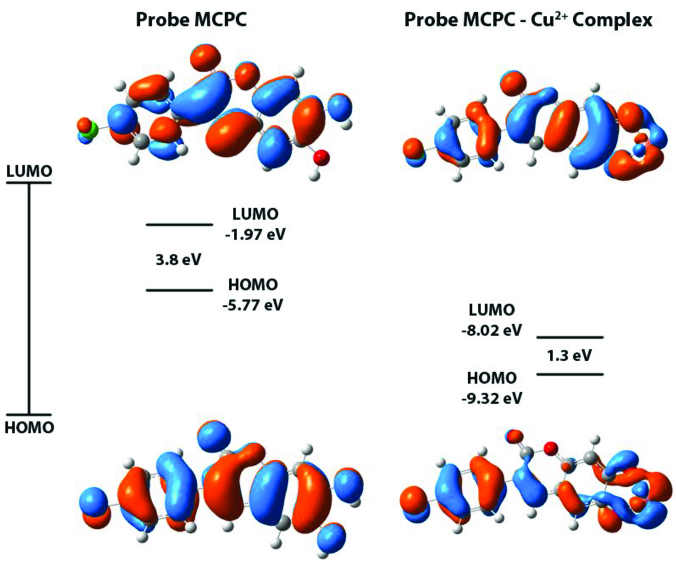
Energy level diagrams of the MCPC-Cu^2+^ complex and probe MCPC.

To determine the fluorometric sensing mechanism of probe MCPC binding to Cu^2+^, theoretical computations were done by using the DFT / B3LYP/6-31 g (d,p) method with Gaussian-09 software package with and accompanying graphical interface program GaussView 5.0.8 [33–37]. The LanL2DZ basis set for the effective potential set for Cu, and the 6-311G basis set was employed for C, H, O atoms (Figure 7). The geometric optimizations were performed in the excited states to obtain the energy minimized structures of probe MCPC and MCPC-Cu^2+^ complex. As presented in Figure 7, the energy gap (ΔE) of probe MCPC was calculated to be 3.80 eV, and after Cu^2+^ addition, the ΔE decreased remarkably to 1.30 eV. We offered the stability of MCPC-Cu^2+^ complex was better than probe MCPC based on the theoretical calculations. The increase in the stability indicates that the route of the reaction tends to the formation of MCPC-Cu^2+^ complex, and therefore the fluorescence quenching occurs.

### 3.4. Fluorometric sensing and ICP-OES analysis of the tea samples

To assess the potential usage of probe MCPC for practical applications, Cu^2+^ levels were measured in various herbal tea samples, as well as black tea samples (Table 2). The samples were analyzed by the calibration plot (Figure 6b) of the emission intensities of probe MCPC toward Cu^2+^. A standard addition method was also employed by adding two different concentrations (0.1 and 0.2 μmol L−1) of Cu^2+^ into the tea samples. As indicated in Table S2, probe MCPC could be very practical for Cu^2+^ sensing with satisfactory accuracy and precision values in tested tea samples. The fluorometric sensing method’s recovery values were calculated within a range from 90.22% to 109.19%. Thus, Cu^2+^ can be identified with excellent recovery values in herbal and black tea samples.

**Table 2:** Determination of Cu^2+^ in herbal and black teas by ICP-OES and probe MCPC.

	Cu^2+^ (μmol.L^−1^)		Difference of the means	Statistics (df = 2)			
	MPCP	ICP-OES		t_Statistic_	probability >|t|
Herbal tea samples						
Green tea	0.1745 ±0.0021	0.1724 ±0.0017	0.0022	2.5476	0.1257	t_tabulated_>t_calculated_
Green tea (mixed with rose)	0.0244 ±0.0004	0.0254 ±0.0006	–0.0009	–1.8199	0.2104	
White tea	0.1267 ±0.0010	0.1276 ±0.0017	–0.0009	–1.2051	0.3514	
Sage tea	0.0689 ±0.0009	0.0682 ±0.0014	0.0006	1.3093	0.3206	
Fennel tea	0.0384 ±0.0004	0.0386 ±0.0007	–0.0001	–0.3780	0.7418	
Daisy tea	0.0791 ±0.0008	0.0789 ±0.0010	0.0002	2.4277	0.1359	
Rose hip tea	0.0664 ±0.0010	0.0662 ±0.0012	0.0003	1.8898	0.1994	
Ginger tea	0.0222 ±0.0002	0.0221 ±0.0011	0.0002	0.3111	0.7852	
Mint tea	0.0851 ±0.0010	0.0855 ±0.0017	–0.0003	–0.4804	0.6784	
Apple tea	0.0104 ±0.0010	0.0102 ±0.0006	0.0002	0.5547	0.6348	
Linden tea	0.0880 ±0.0009	0.0876 ±0.0013	0.0004	0.7184	0.5471	
Black tea samples						
Black tea without aroma-A	0.0843 ±0.0010	0.0845 ±0.0022	–0.0002	–0.2857	0.8019	t_tabulated_ > t_calculated_
Black tea without aroma-B	0.0165 ±0.0002	0.0169 ±0.0006	–0.0004	–1.7320	0.2254	
Black tea without aroma-C	0.0535 ±0.0004	0.0537 ±0.0013	–0.0002	–0.3255	0.7757	
Black tea with bergamot aroma-A	0.1321 ±0.0009	0.1319 ±0.0031	0.0002	0.1644	0.8845	
Black tea with bergamot aroma-B	0.0506±0.0003	0.0522 ±0.0021	–0.0015	–1.4034	0.2956	

†mean(1)-mean(2) = 0 based on Null Hypothesis; mean(1)-mean(2) <>0 based on Alternative Hypothesis; the differentiation of the means is NOT considerably dissimilar with the test variation (0) (at the 0.05 level).

The validity of the proposed sensing method for Cu^2+^ detection was checked by employing ICP-OES analysis. Likewise the proposed fluorescence method, samples were spiked with recognized quantities of Cu^2+^ (0.01–0.02 mgL^−1^) (Table S3). The calibration graphs were constructed for the determination of Cu^2+^ quantitatively (0.01–1.00 mgL^−1^) by ICP-OES method. Recoveries of Cu^2+^ were between 94.58 and 105.66% values, and the good agreements were found between the obtained and added quantities of Cu^2+^. The results of the fluorescence and atomic spectroscopy methods were compared (Table 2), and the differences of the means of two methods were not found significantly different from each others at the 0.05 level.

## Conclusion

As a conclusion, we have developed a low-cost, highly selective and sensitive probe MCPC with quite-low detection limit for the fluorescent sensing of Cu^2+^. Probe MCPC demonstrated excellent fluorescence “turnoff” response to Cu^2+^ in CH_3_CN/H_2_O media (v/v, 95/5, 5.0 μM) at 456 nm with emission quenching, and it revealed a rapid response time (~60 s). The detection limit was found as 1.47 nM according to 3σ/slope, which indicated excellent sensitivity to Cu^2+^ sensing in nanomolar levels. The ΔE values of the probe MCPC and its complex were calculated to be 3.80 eV and 1.30 eV, respectively. Studies on real samples, herbal and black teas, revealed that the probe MCPC can sensitively and selectively recognize Cu^2+^ with good recovery values (90.22%–109.19%). The outcomes of the probe MCPC and ICP-OES analyses were also compared and the differences of the means of two methods were not found significantly different (P <0.05). Thus, these promising findings could make a great contribution to researchers studying Cu^2+^ detection in different foodstuffs.

Supplementary Materials
