# Supplementary material for: Fluorescent sensing platform for low-cost detection of Cu2+ by coumarin derivative: DFT calculation and practical application in herbal and black tea samples
Source: Turk J Chem. 2020 Aug 18;44(4):1148–63. doi: 10.3906/kim-2004-63 (PMC7751904; doi:10.3906/kim-2004-63)
Supplement: Supplementary file 1 — Supplementary Materials [file turkjchem-44-1148-sup001.pdf]

Supplementary Information

MATERIALS AND METHODS

Microwave–assisted solvent free synthesis of probe MCPC

3.03 mmol Phenylacrylonitrile compound **(3)** [1–3] and pyridinium hydrochloride (5 g) were reacted in silica gel and the reaction mixture was subjected to microwave irradiation at 320 watt for 30 min. The reaction was quenched with 1.0 N HCl (200 mL) as soon as the reaction completed. The reaction mixture filtered, the residue dissolved in acetone (20 mL) four times and filtered over silica gel. The solvent was evaporated and the residue washed with water and then dried. The obtained solid was precipitated with ethyl acetate (15 mL) and n–hexane (250 mL). The obtained crude product was separated by column chromatography.

A fawn colored solid compound, probe **MCPC**, was obtained (0.75 g, 85%). Anal. Calc. for $C_{15}H_9ClO_4$ (MW: 288.68): C, 62.41; H, 3.14; Found C, 62.44, H; 3.17%. FT–IR (KBr, cm^{-1}): 3197, 3398$\nu_{O–H}$, 3000–3090 $\nu_{C–H(Ar)}$, 1660 $\nu_{C=O}$, 1563, 1571, and 1621 $\nu_{C=C}$. ^1H NMR (400 MHz, DMSO–d_6): δ 6.80 (1H, s, H^2), 7.08 (1H, s, H^5), 7.51 (2H, d, *J*=8.4 Hz, H^{13}), 7.74 (2H, d, *J*=8.8 Hz, H^{14}), 8.16 (1H, s, H^9), 9.56 (1H, s, H^7), 10.34 (1H, s, H^8). ^{13}C–NMR (400 MHz, DMSO–d_6): δ 143.59 C^1, 102.69 C^2, 151.21 C^3, 148.65 C^4, 112.92 C^5, 111.88 C^6, 141.92 C^9, 121.27 C^{10}, 160.66 C^{11}, 132.97 C^{12}, 128.59 $C^{13,17}$, 130.48 $C^{14,16}$, and 134.63 C^{15}.

RESULTS AND DISCUSSION

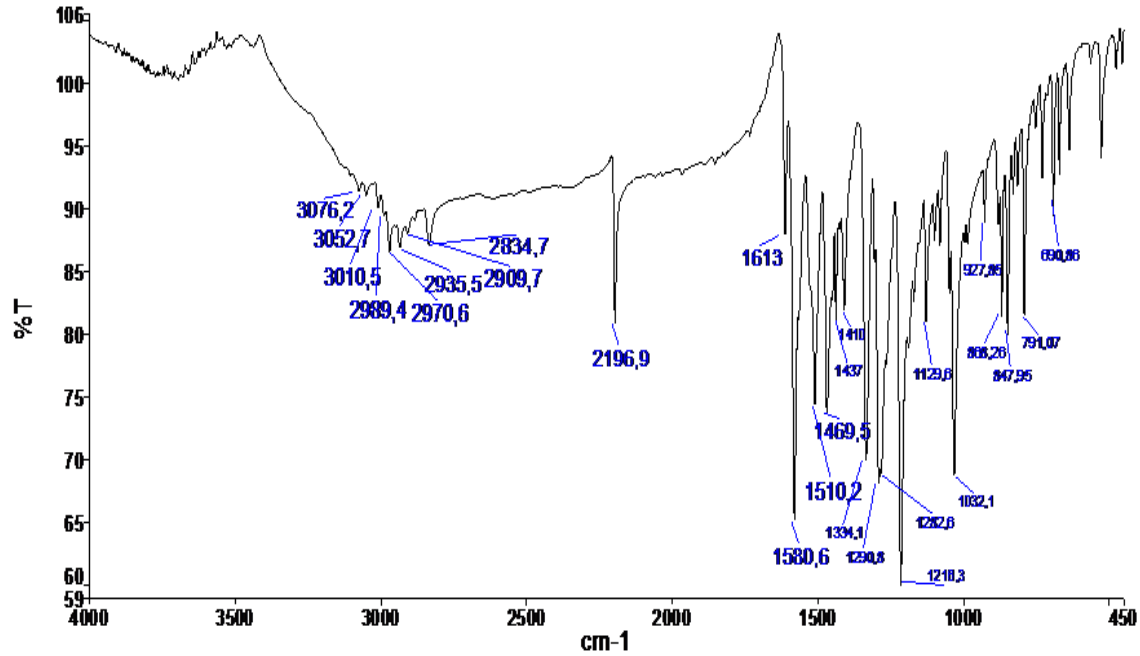

Figure S1. FT–IR spectrum of the compound **(3)**

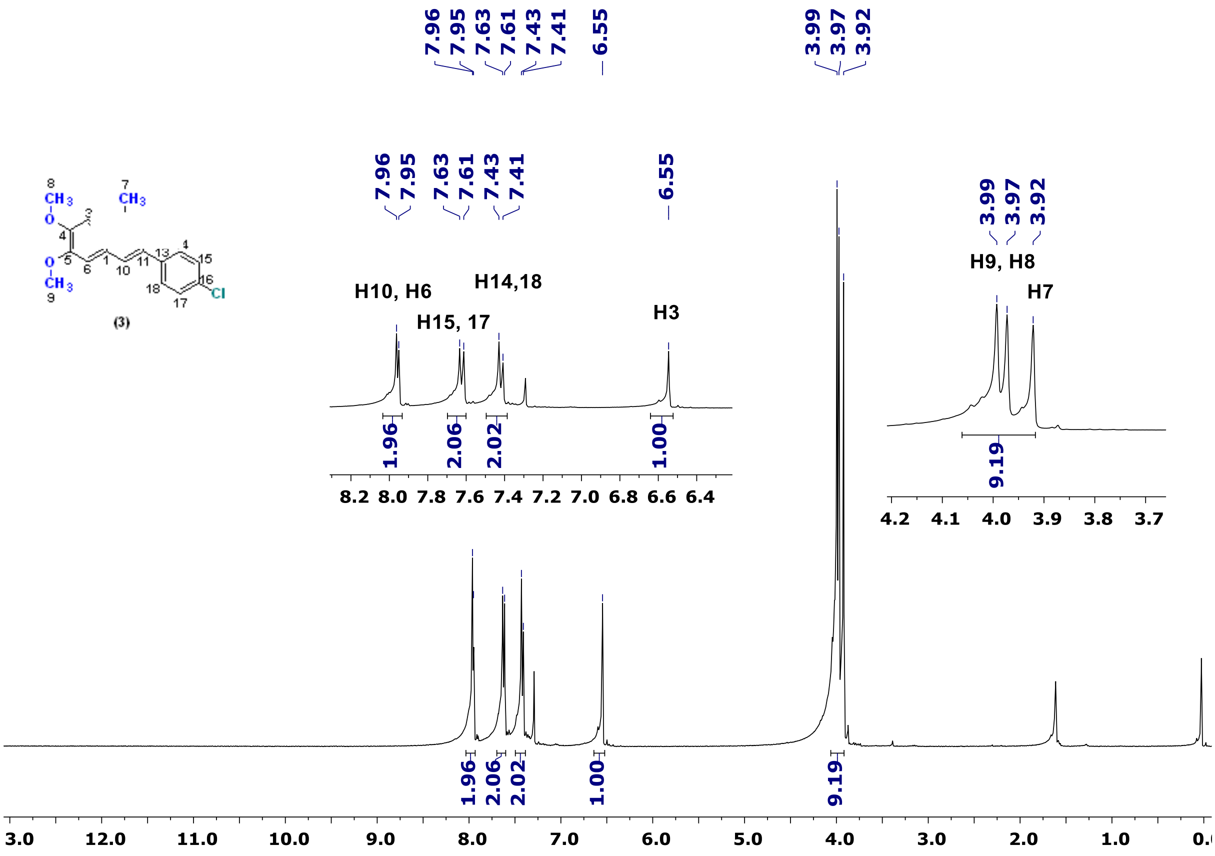

Figure S2. ^{1}H NMR spectrum of the compound **(3)** in $CDCl_3$–d_1

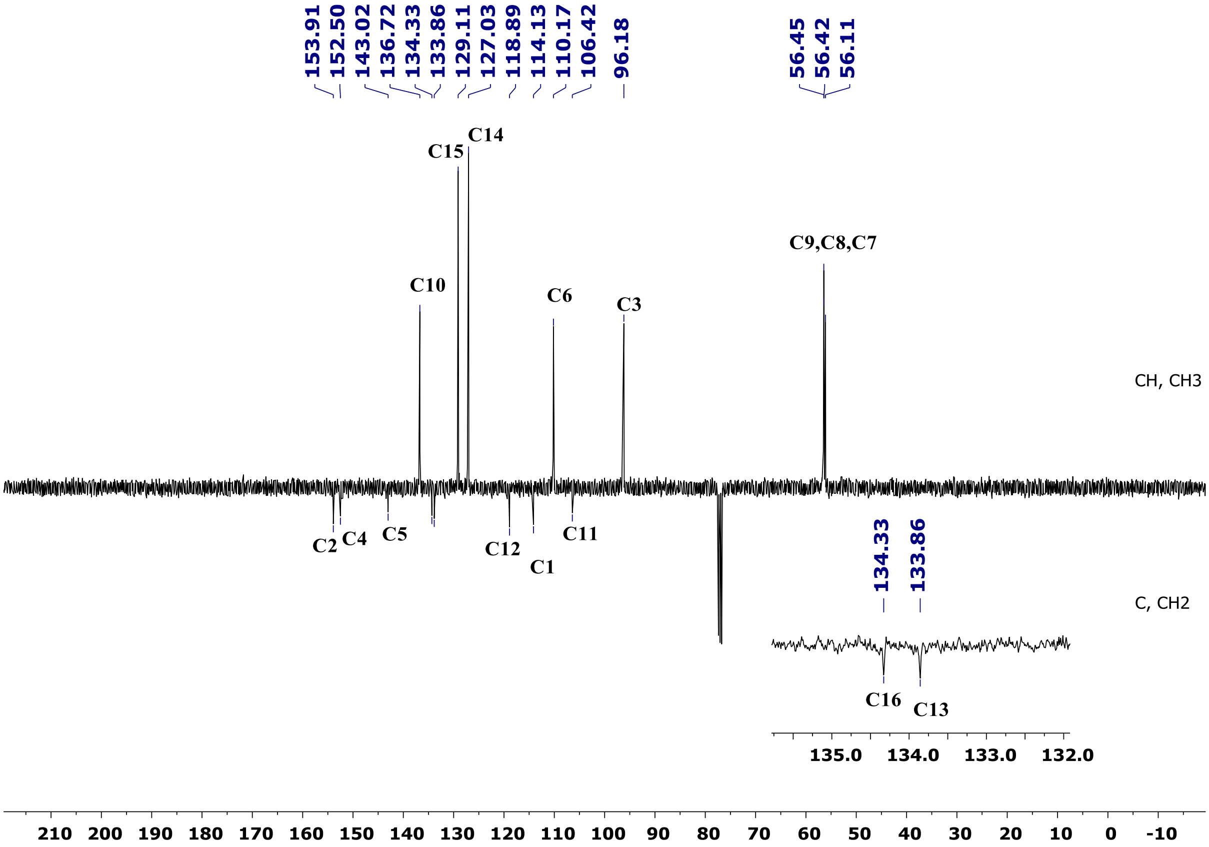

Figure S3. ^{13}C–APT NMR spectrum of the compound **(3)** in $CDCl_3$–d_1

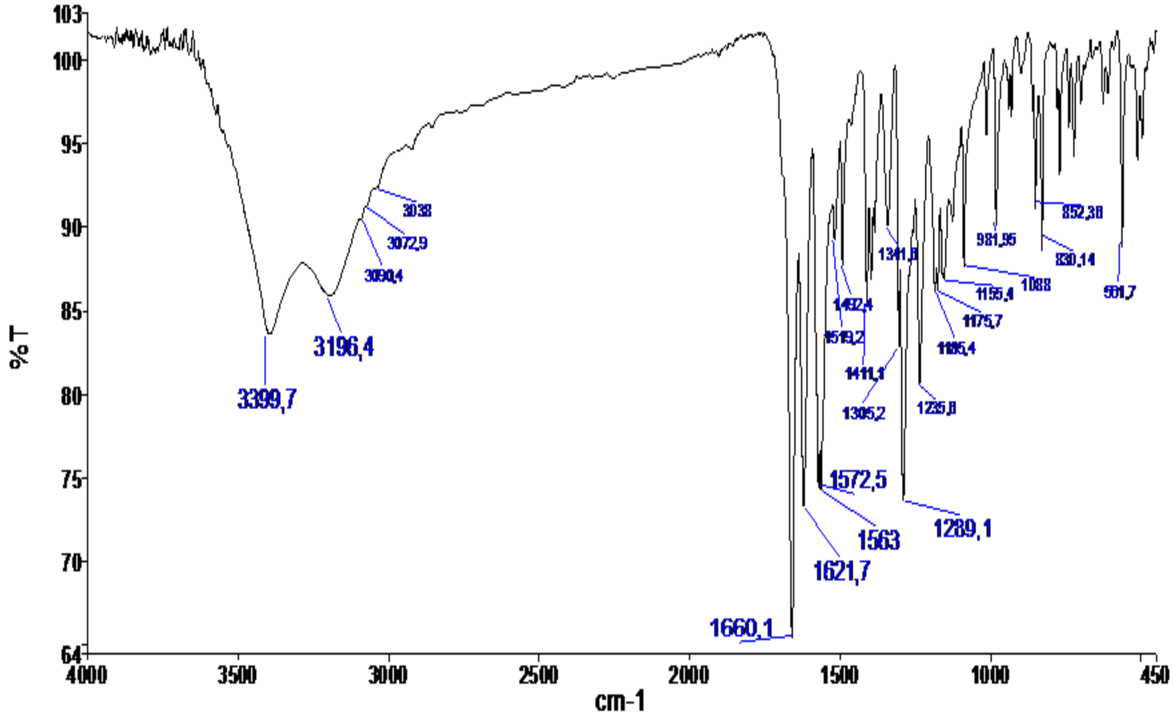

Figure S4. FT–IR spectrum of the probe **MCPC**

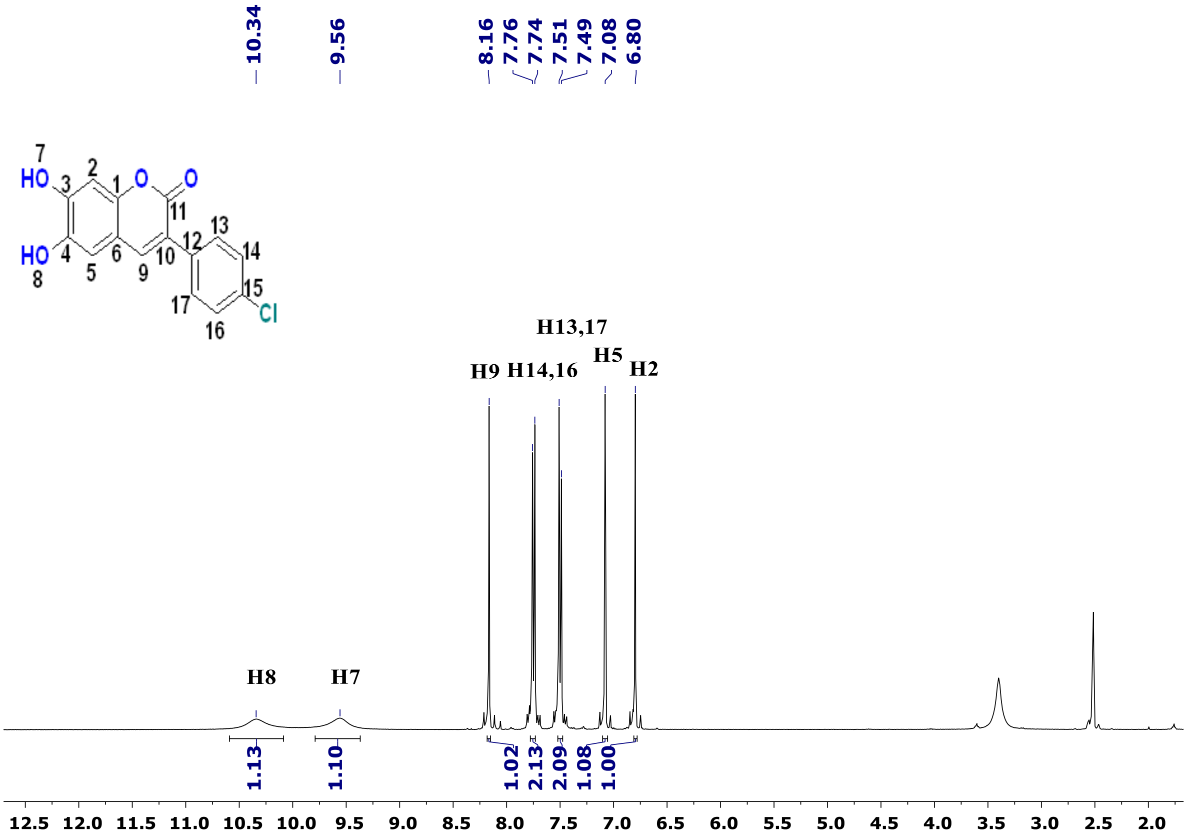

Figure S5. ^{1}H NMR spectrum of the probe **MCPC** in DMSO–d_6

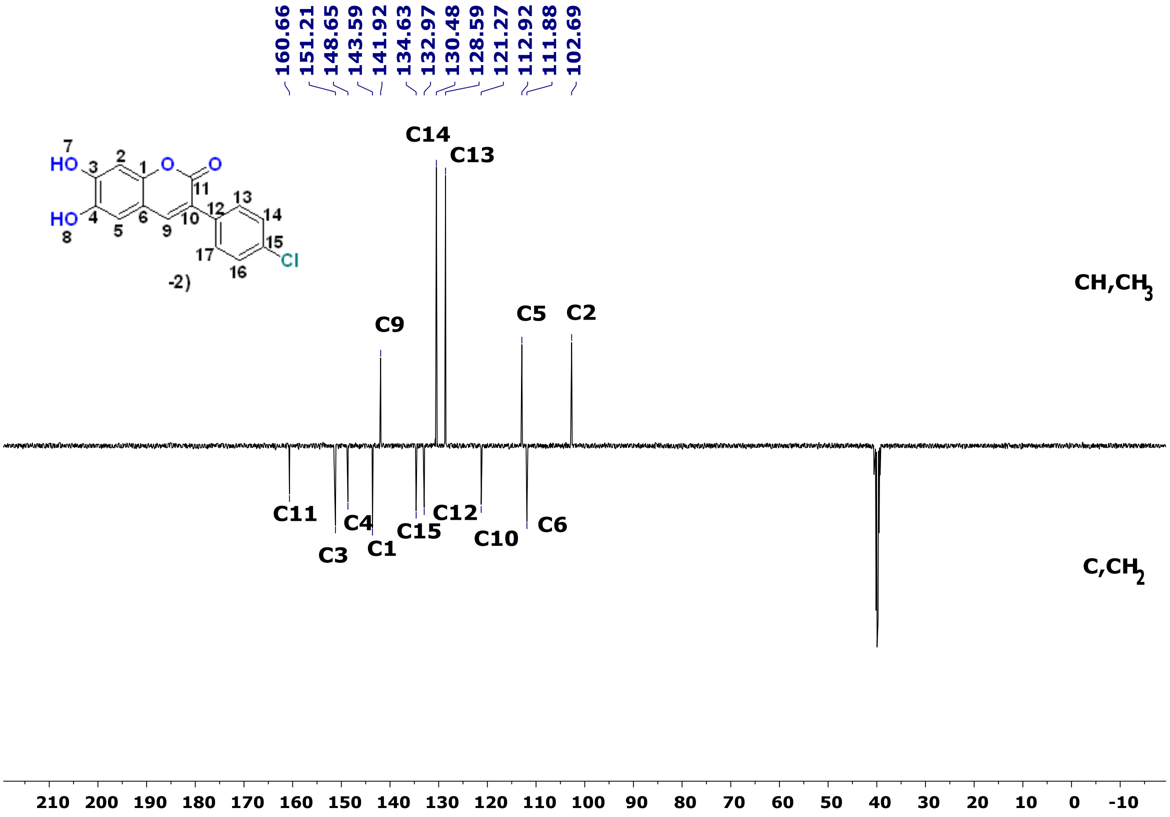

Figure S6. ^{13}C–APT NMR spectrum of the probe **MCPC** in DMSO–d_6

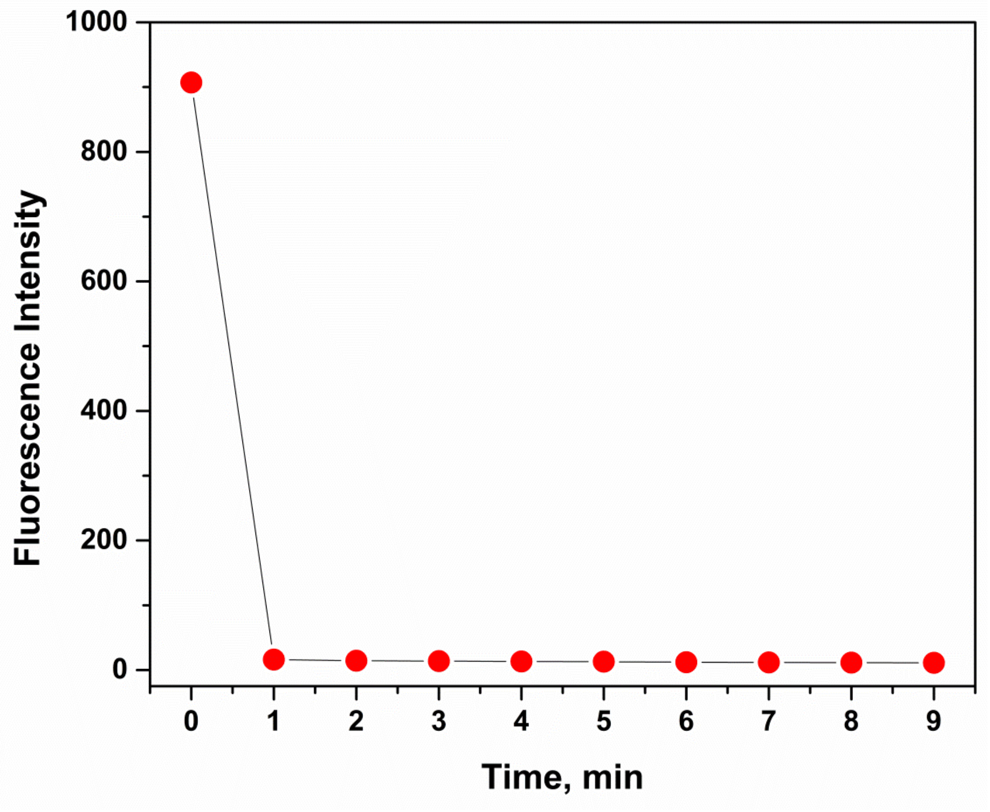

Figure S7. Response time of the **MCPC–Cu^{2+}** complex

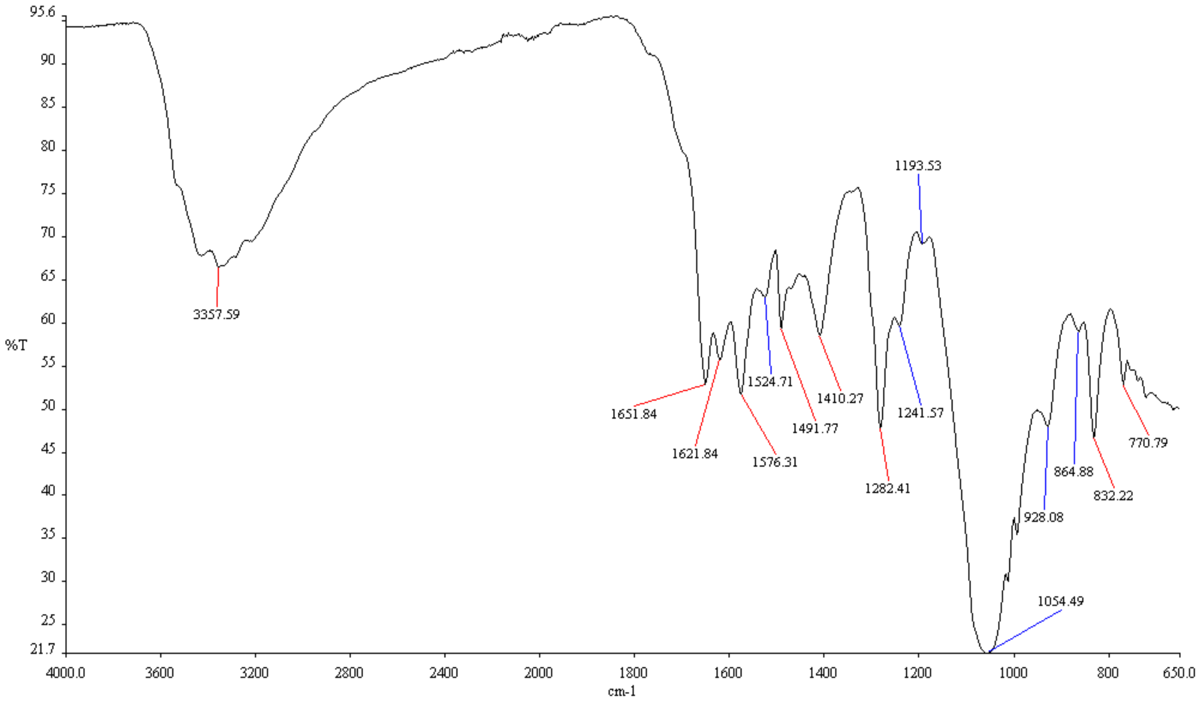

Figure S8. FT–IR spectrum of the **MCPC–Cu^{2+}** complex

Table S1. Operating parameters for the ICP–OES analysis

parameter	***setting***
Plasma torch:	standard one piece quartz axial, one–piece with 2.4 mm injector
Spray chamber type:	glass cyclonic (single–pass)
Supplied rf power:	1.0 kW
Plasma Ar gas flow:	10 $L.min^{-1}$
Auxiliary Ar gas flow:	1.0 $L.min^{-1}$
Nebulizer Ar gas flow:	0.85 $L.min^{-1}$
Nebulizer type:	sea spray
Pump tubing Rinse/Instrument pump:	white–white tabs (1.02 mm id) – Waste: blue–blue tabs (1.65 mm id)
Pump speed:	20 rpm
Total sample usage:	1 mL
Replicate read time:	15 sec
Number of replicates:	5
Sample uptake delay time:	20 sec
Stabilization time:	10 sec
Rinse time:	10 sec
Fast pump:	on
Wavelengths from 167 to 785 nm	Cu 324.754 nm, Cd 228.8 nm, Hg 253.7 nm, Zn 213.8 nm, Pb 405.8 nm, Ag 328.1 nm, and Tl 377.6 nm

Table S2. Determination of Cu^{2+} in herbal and black tea samples by the probe **MCPC**

sample	Cu^{2+} added (µmol L^{-1})	Cu^{2+} found (µmol L^{-1})	recovery (%)	RSD (%) (n=3)
herbal tea samples				
green tea	0.00	0.17 ±0.002		1.20
	0.10	0.27 ±0.003	100.65	0.92
	0.20	0.37 ±0.002	99.28	0.56
green tea (mixed with rose)	0.00	0.02 ±0.0004		1.75
	0.10	0.12 ±0.002	91.64	1.31
	0.20	0.23 ±0.003	104.42	1.38
white tea	0.00	0.13 ±0.001		0.80
	0.10	0.22 ±0.002	93.41	0.90
	0.20	0.32 ±0.002	99.52	0.81
sage tea	0.00	0.07 ±0.0001		1.22
	0.10	0.16 ±0.001	93.63	0.61
	0.20	0.29 ±0.002	108.23	0.73
fennel tea	0.00	0.04 ±0.0004		1.04
	0.10	0.13 ±0.001	90.22	0.59
	0.20	0.24 ±0.003	101.25	0.98
daisy tea	0.00	0.08 ±0.001		1.06
	0.10	0.18 ±0.003	98.36	1.41
	0.20	0.27 ±0.004	96.10	1.29
rose hip tea	0.00	0.07 ±0.001		1.48
	0.10	0.17 ±0.002	105.78	0.88
	0.20	0.25 ±0.003	91.70	1.00
ginger tea	0.00	0.02 ±0.0002		1.03
	0.10	0.11 ±0.001	91.79	0.88
	0.20	0.22 ±0.001	98.73	0.50
mint tea	0.00	0.09 ±0.001		1.16
	0.10	0.19 ±0.001	102.17	0.27
	0.20	0.27 ±0.004	93.16	1.57
apple tea	0.00	0.01 ±0.0001		0.97
	0.10	0.12 ±0.002	106.51	1.31
	0.20	0.21 ±0.001	101.06	0.47
linden tea	0.00	0.09 ±0.001		1.07
	0.10	0.20 ±0.001	109.19	0.50
	0.20	0.30 ±0.002	104.17	0.51
black tea samples				
black tea without aroma–A	0.00	0.08 ±0.001		1.19
	0.10	0.19 ±0.001	100.80	0.40
	0.20	0.29 ±0.001	102.63	0.35
black tea without aroma–B	0.00	0.02 ±0.0002		1.38
	0.10	0.12 ±0.002	100.82	1.28
	0.20	0.23 ±0.003	108.37	1.14
black tea without aroma–C	0.00	0.05 ±0.0002		0.65
	0.10	0.14 ±0.002	90.47	1.39
	0.20	0.27 ±0.003	108.92	0.93
black tea with bergamot aroma–A	0.00	0.13 ±0.001		0.65
	0.10	0.23 ±0.002	101.35	0.65
	0.20	0.34 ±0.003	104.06	0.90
black tea with bergamot aroma–B	0.00	0.05 ±0.0003		0.53
	0.10	0.15 ±0.001	99.55	0.77
	0.20	0.23 ±0.003	91.02	1.29

Table S3. Determination of Cu^{2+} in herbal and black tea samples by **ICP–OES**

sample	Cu^{2+} added (μg L^{-1})	Cu^{2+} found (μg L^{-1})	recovery (%)	RSD (%) (n=3)
herbal tea samples				
green tea	0	10.96 ±0.11		0.96
	10	20.55 ±0.16	95.96	0.78
	20	30.72 ±0.13	98.83	0.43
green tea (mixed with rose)	0	1.16 ±0.04		2.44
	10	11.46 ±0.10	98.77	0.85
	20	21.67 ±0.38	100.27	1.74
white tea	0	8.11 ±0.11		1.35
	10	17.88 ±0.112	97.67	0.70
	20	27.76 ±0.17	98.28	0.61
sage tea	0	4.34 ±0.09		1.98
	10	14.45 ±0.06	101.11	0.44
	20	24.23 ±0.13	99.48	0.55
fennel tea	0	2.45 ±0.05		1.96
	10	12.16 ±0.05	97.08	0.40
	20	22.48 ±0.15	100.14	0.67
daisy tea	0	5.02 ±0.06		1.28
	10	15.15 ±0.16	101.39	1.05
	20	25.52 ±0.22	102.50	0.87
rose hip tea	0	4.21 ±0.08		1.87
	10	14.11 ±0.10	99.08	0.69
	20	24.42 ±0.46	101.09	1.88
ginger tea	0	1.40 ±0.07		4.97
	10	11.25 ±0.06	98.52	0.56
	20	20.90 ±0.07	97.50	0.33
mint tea	0	5.43 ±0.11		2.00
	10	16.00 ±0.03	105.66	0.20
	20	25.39 ±0.27	99.81	1.07
apple tea	0	0.65 ±0.04		5.76
	10	10.44 ±0.10	97.97	0.93
	20	19.56 ±0.06	94.58	0.32
linden tea	0	5.57 ±0.08		1.48
	10	15.59 ±0.06	100.27	0.41
	20	24.87 ±0.10	96.53	0.39
black tea samples				
black tea without aroma–A	0	5.37 ±0.14		2.68
	10	14.82 ±0.05	94.46	0.32
	20	25.34 ±0.06	99.84	0.25
black tea without aroma–B	0	1.08 ±0.04		3.35
	10	11.44 ±0.10	103.66	0.83
	20	20.93 ±0.17	99.30	0.80
black tea without aroma–C	0	3.41 ±0.08		2.43
	10	13.30 ±0.13	98.84	0.96
	20	23.50 ±0.30	100.43	1.29
black tea with bergamot aroma–A	0	8.38 ±0.19		2.32
	10	17.91 ±0.10	95.33	0.54
	20	27.78 ±0.19	97.01	0.70
black tea with bergamot aroma–B	0	3.31 ±0.13		3.90
	10	13.46 ±007	101.41	0.54
	20	23.73 ±0.26	102.05	1.08

References

1. Ozen F, Tekin S, Koran K, Sandal S, Gorgulu AO. Synthesis, structural characterization, and in vitro anti-cancer activities of new phenylacrylonitrile derivatives. Applied Biological Chemistry 2016; 59 (1): 239-248. doi: 10.1007/s13765-016-0163-x
2. Buu-Hoi NP, Saint-Ruf G, Lobert B. Oxygen heterocycles. part XIV. hydroxylated 3-awl- and 3-pyridyl-coumarins. Journal of the Chemical Society C: Organic 1968; 16 (1): 2069-2070. doi: 10.1039/J39690002069
3. Elgazzar E, Dere A, Özen F, Koran K, Al-Sehemi AG et al. Design and fabrication of dioxyphenylcoumarin substituted cyclotriphosphazene compounds photodiodes. Physica B: Condensed Matter 2017; 515 (1): 8-17. doi: 10.1016/j.physb.2017.03.025
